# Trompe l’oeil electrocardiogram

**DOI:** 10.1007/s12471-017-0953-8

**Published:** 2017-01-18

**Authors:** E. Ströker, C. de Asmundis, G. B. Chierchia, P. Brugada

**Affiliations:** Heart Rhythm Management Centre, University Hospital Brussels, Free University of Brussels, Brussels, Belgium

## Answer

**Fig. 1:**
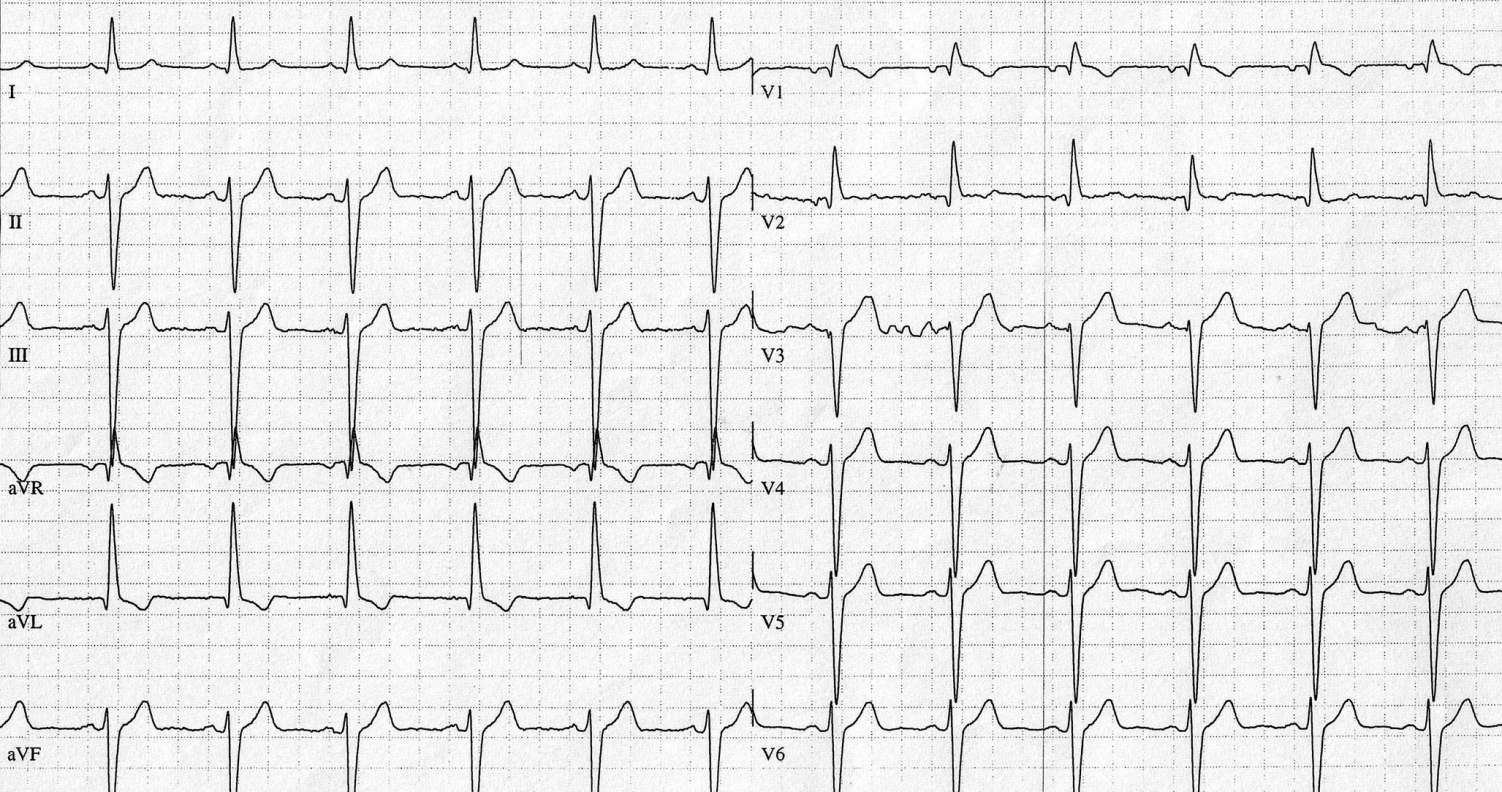
Unipolar mapping of the right precordial leads (V1, V2) one intercostal space higher (IC 3) uncovering the classic pattern of a complete RBBB. In contrast, note the total suppression of RBBB signs (S waves in limb leads I, aVL; terminal R wave in V1) looking back at figure 1 in the question, where the ECG leads were placed conventionally

Given the QRS width, the intraventricular conduction de﻿lay could not be explained solely by the left anterior hemiblock (LAHB). Placement of the rightward precordial leads V1–V2 one intercostal (IC) space above the conventional level uncovered a terminal R‑wave suggestive of complete right bundle branch block (RBBB) (Fig. 1). Although there might be a contributing role for delayed activation of the anterobasal left ventricle wall in explaining this R’ wave (not determined), the electrophysiological study was in favour of a proximal RBBB (QRS to right ventricle apex interval of 36 ms), and showed a high risk for atrioventricular block: baseline His-ventricular interval of 57 ms, prolonging to 102 ms with some infra-Hisian blocked atrial pacing beats (CL 420 ms) under ajmaline. No structural or ischaemic heart disease was revealed. Considering these findings, the use of a class Ic antiarrhythmic drug was most likely the cause of the paroxysmal atrioventricular block in our patient. An implantable loop recorder could have been considered in our patient as propafenone had already been stopped, but we decided to give prophylactic double chamber pacing therapy. There was no recurrence of brady-symptomatology during follow-up, but palpitations were still noted with episodes of paroxysmal atrial fibrillation seen in the memory of the device, for which ablation therapy was provided.

Although already described in 1973, the entity of a masquerading RBBB remains intriguing [1]. Baseline ECG showed a normal initial vector for LAHB, however, the main vector being extremely superior (deep S waves in inferior leads and V6), left, and more posterior than usual (negative precordial concordance). It seems that powerful vertical forces can provoke a QRS change only by a small displacement of the chest electrodes. Below the transition point, mapped between IC4 and IC3, the left and posterior forces of the LAHB seemed important and late enough to suppress the right anterior terminal forces of the RBBB. As other aetiologies of leftward terminal forces could be ruled out, a high-grade LAHB is the most plausible explanation for this phenomenon in our patient. This diagnosis is important as failure to recognise it can cause clinical error.
